# The Seattle Heart Failure Model in Kidney Transplant Recipients

**DOI:** 10.3390/jcm12247614

**Published:** 2023-12-11

**Authors:** Angelica Perez-Gutierrez, Rita L. McGill, Braden Juengel, Piotr J. Bachul, David N. Danz, Michelle Josephson, Ben B. Chung, Ann Nguyen, John J. Fung, Rolf N. Barth, Yolanda T. Becker

**Affiliations:** 1 Department of Surgery, Transplant Institute, University of Chicago, Chicago, IL 60637, USA; 2 Section of Nephrology, Department of Medicine, University of Chicago, Chicago, IL 60637, USA; 3 Department of Economics, University of Pittsburgh, Pittsburgh, PA 15260, USA; 4 Section of Cardiology, Department of Medicine, University of Chicago, Chicago, IL 60637, USA

**Keywords:** kidney transplant, cardiac risk, mortality, Seattle Heart Failure Model

## Abstract

Cardiovascular disease is the leading cause of mortality following kidney transplantation. Heart failure affects 17–21% of patients with chronic kidney disease and increases along with time receiving dialysis. The Seattle Heart Failure Model (SHFM) is a validated mortality risk model for heart failure patients that incorporates clinical, therapeutic, and laboratory parameters but does not include measures of kidney function. We applied the SHFM to patients with end-stage renal disease (ESRD) who were being evaluated for kidney transplantation to determine if the model was associated with post-transplant mortality. This retrospective single-center study analyzed survival among 360 adult deceased-donor kidney transplant recipients. Cox regression was used to model post-transplant patient survival. Our findings indicated that a 1.0-point increase in the adapted SHFM score was significantly associated with post-transplant mortality (HR 1.76, 95% CI = 1.10–2.83, *p* = 0.02), independently of the Kidney Donor Profile Index and Estimated Post-Transplant Survival. Individual covariates of the SHFM were evaluated in univariate analyses, and age, sodium, cholesterol, and lymphocyte count were significantly related to mortality. This study provides preliminary evidence that an adapted SHFM score could be a useful tool in evaluating mortality risk post-transplant in patients with ESRD.

## 1. Introduction

Cardiovascular disease accounts for 40 to 60% of all deaths after kidney transplantation [1,2]. This association reflects shared risk factors between kidney failure and cardiovascular disease, such as diabetes and hypertension. Moreover, severe chronic kidney disease can exacerbate cardiac disease due to fluid overload, mineralocorticoid excess, anemia, uremic toxins, and soft-tissue calcifications that contribute to atherosclerosis, coronary artery disease, left-ventricular hypertrophy, and decreased coronary perfusion [3].

Heart failure occurs in 17–21% of patients with chronic kidney disease, and its incidence increases with the duration and severity of the disease [4]. Heart failure episodes occur in approximately 44% of patients receiving maintenance hemodialysis [5]. The limited supply of suitable organs means that most patients wait for years before receiving transplants, during which cardiovascular risks increase. The prevalence of heart failure among patients on the transplant waiting list increases over time, from 12% at one year to 32% at three years [6].

Most existing mortality risk models incorporate measures of kidney function as covariates, complicating the ability to evaluate the impact of cardiovascular risk factors on mortality in kidney failure patients [7,8]. The Seattle Heart Failure Model (SHFM) is an already-validated mortality risk model that does not utilize kidney function biomarkers. The SHFM uses age, gender, New York Heart Association (NYHA) class, ejection fraction (EF), ischemic disease, systolic blood pressure, angiotensin-converting enzyme inhibitor (ACEI), angiotensin receptor blocker (ARB), statin, diuretics, allopurinol, presence of an implantable cardioverter defibrillator (ICD), sodium, hemoglobin, lymphocytes, uric acid, and cholesterol, with coefficients derived from major cardiovascular trials. A revised version was later introduced without the statin variable. The SHFM has been widely validated as a prognostic tool for patients with heart failure and an ejection fraction <40% [9]. The SHFM has also been tested in patients with heart failure and preserved EF, although its predictive ability is slightly less in these patients [10]. Due to shared risk factors between heart and renal disease, the SHFM incorporates several known risk factors for mortality in patients with end-stage renal disease (ESRD) or kidney transplants [11,12,13,14,15,16,17]. 

Candidates for kidney transplantation undergo rigorous cardiac evaluations, including echocardiography, stress tests, and cardiac catheterization, to ensure good cardiac function; symptomatic heart failure is a contraindication to transplant. Despite this comprehensive evaluation, the high prevalence of cardiovascular disease including heart failure post-transplant indicates a need for better risk stratification tools in this patient population.

We sought to determine whether a weighted clinical risk model such as the SHFM could provide a non-invasive and cost-effective approach to refining the preoperative assessment of cardiovascular risk. We therefore applied an adaptation of the SHFM risk score to patients with ESRD at the time of their kidney transplant evaluation and analyzed associations with post-transplant mortality.

## 2. Patients and Methods

This was a single-center retrospective study of deceased-donor kidney transplants performed at the University of Chicago Medical Center from 2012–2020. Pediatric and multiple organ transplants were excluded. 

Clinical and biochemical data were primarily obtained from the electronic medical records available at the time of the most recent pre-transplant evaluation. Ischemia was defined as a positive history of myocardial infarction or coronary artery disease. History of heart failure was obtained from the medical records. Echocardiography was performed during the transplant evaluation; in our program, patients with LV-EF < 30% are not considered for kidney transplant alone and are referred to the heart failure clinic. The lymphocyte count was the only variable assessed during the 24 h prior to transplant surgery. 

Mean and median values were used to summarize normally and non-normally distributed continuous covariates. Categorical variables were expressed as percentages, with t-tests, Mann–Whitney U tests, chi-squared tests, and Fisher exact tests used to compare groups where appropriate. Missing data for variables required for calculation of the adapted SHFM were handled by multiple imputation using the Markov-chain Monte Carlo method, with generation of 25 imputation data sets. A *p*-value < 0.05 was considered statistically significant.

Our adaptation of the SHFM score was derived from the original report of the SHFM [9] and the online updated calculator https://depts.washington.edu/shfm (accessed on 1 August 2022) Most of the updated variables (IV diuretics, inotropes, intra-aortic balloon pumps, ventilators, continuous ultrafiltration, and devices such as left ventricular assist devices and defibrillators) were left out because these are not applicable to kidney transplant candidates. We removed the variable for HMG-CoA reductase inhibitors (statins) because this variable was eventually removed in the updated SHFM “given the negative results with the addition of statins in large randomized clinical trials”. We validated our calculation against an example given in the original work of Levy et al. [9]. Further details are available in the supplementary data. 

The Kidney Donor Profile Index (KDPI) was obtained from DonorNET. The Expected Post-Transplant Survival (EPTS) score was calculated from data available at the time of transplant, using methods described elsewhere [18]; raw EPTS scores were used to eliminate interpolation based on year of transplant.

Time zero for survival models was set as the date of transplant surgery, to eliminate immortal time bias. Subjects were followed until death or 30 December 2021, whichever came first. Univariate Cox proportional hazards models were constructed to obtain hazard ratios for death after transplantation, including each component of the SHFM score and several transplant-specific variables. We developed a series of multivariable Cox models to determine whether inclusion of KDPI, EPTS score, or other kidney transplant specific variables were independent predictors of post-transplant mortality. The Akaike Information Criterion was used to determine the model with optimal goodness of fit, and Harrell’s c-index was calculated as a measure of discrimination for this model. The univariate model was used to generate predicted survival as a function of SHFM scores in kidney transplant recipients and was compared to predicted survival in heart failure patients, and the optimal model was used to create a prediction nomogram for survival at two years and five years after transplantation. 

As a sensitivity analysis, we repeated our calculations with the statin variable restored to the model. All analyses were conducted using SAS, version 9.4 (SAS Institute Inc., Cary, NC, USA) and the “survival” and “rms” packages from R, version 4.2.2 (R Foundation for Statistical Computing, Vienna, Austria).

## 3. Results

### 3.1. Demographic and Clinical Data

A total of 360 patients were analyzed. Median follow up was 46 months (interquartile range (IQR) = 23–76 months). Mean age at the time of evaluation for transplant was 51 years; 64% were male, and 69% were African American. 

The main causes of kidney disease were hypertension (28.6%) and diabetes (25.8%).

The median wait-time between the last evaluation and the transplant was 9.5 (IQR = 4.6–16.4) months. Median exposure to dialysis before transplant was 72 (IQR = 47–90) months, and only 18 patients (5%) were transplanted pre-emptively. Table 1 shows the comparison of patient characteristics between surviving and non-surviving patients.

Delayed graft function was present in 40.8% of cases, with no statistically significant difference between patients who survived and those who died (40.1% vs. 46.5%, *p* = 0.4). The incidence of biopsy-proven rejection was 13.9%, also without a statistically significant difference between survivors and non-survivors (13.3% vs. 18.6%, *p* = 0.3).

Death occurred in 43/360 (11.9%) patients over the course of the study. The one-year mortality was 3.6% and the five-year mortality was 9.7%. There were nineteen fatal infections, seven overt cardiac events, and two fatal strokes. The cause of death could not be determined for 15 patients. The median time to death was 20 (IQR = 11–41) months among those who died. The mean EPTS raw score was 2.0 ± 0.7 points among those who survived and 2.4 ± 0.6 points among the patients who died (*p* < 0.001). Compared to survivors, patients who died were older, had higher EPTS, and received allografts with higher KDPI.

### 3.2. Association of the Seattle Heart Failure Model with Post-Transplant Mortality

Compared to survivors, recipients who died had significantly higher SHFM scores (0.16 vs. −0.04, *p* = 0.04). Calculated SHFM scores were roughly normally distributed, with a range of −1.31 to 1.90 (Supplementary Figure S1). 

To determine whether any of the SHFM individual risk factors alone were associated with mortality, we evaluated each of the components with univariate Cox proportional hazards models. Age, sodium, cholesterol, and lymphocyte percentage measured before transplant were significantly associated with mortality after transplant (Table 2).

The distribution of the SHFM and its components between surviving and non-surviving patients are shown in Table 3.

The SHFM was recalculated with the statin variable restored, giving 149 patients who were receiving statins an additional β-coefficient of −0.46, shifting the overall distribution of the SHFM scores leftwards by approximately 0.15 points. The addition of the statin coefficient resulted in reduced fit in both the univariate and multivariable models, by AIC and −2 log L, although the size and directionality of the variable was overall similar to the main model.

### 3.3. SHFM and Other Transplant-Specific Indexes

KDPI and EPTS are transplant specific indices that were built and validated specifically to evaluate deceased kidney donors and transplant recipients. In our cohort, KDPI and EPTS were significantly associated with mortality. Specifically, the univariate hazard ratio for a 10 percent increase in KDPI was 1.25 (95% CI 1.08, 1.45, *p* = 0.004), and each 0.5-point increase in EPTS was associated with a hazard ratio of 1.57 (95% CI 1.23, 2.00, *p* < 0.001). 

The adapted SHFM score was associated with mortality independently of KDPI and EPTS. The unadjusted hazard ratio for a 1.0-point increase in SHFM was 1.76 (95% CI 1.10, 2.83, *p* = 0.02). The best and most parsimonious multivariable model was a three-variable model with SHFM (HR 1.75, 95% CI = 1.06, 2.89, *p* = 0.03), KDPI (HR = 1.17, 95% CI = 1.01, 1.36, per 10% increase, *p* = 0.04), and the EPTS score (HR 1.44, 95% CI = 1.11, 1.87, per 0.5-point increment in raw score, *p* = 0.006). All three variables were independently significant. Further adjustment for other transplant-related variables (panel reactive antibody, donation after circulatory death, organ perfusion) did not improve model performance. There were no significant interactions between the three variables in this model. Harrell’s c-index for this three-variable model was 0.71.

**Figure 1:**
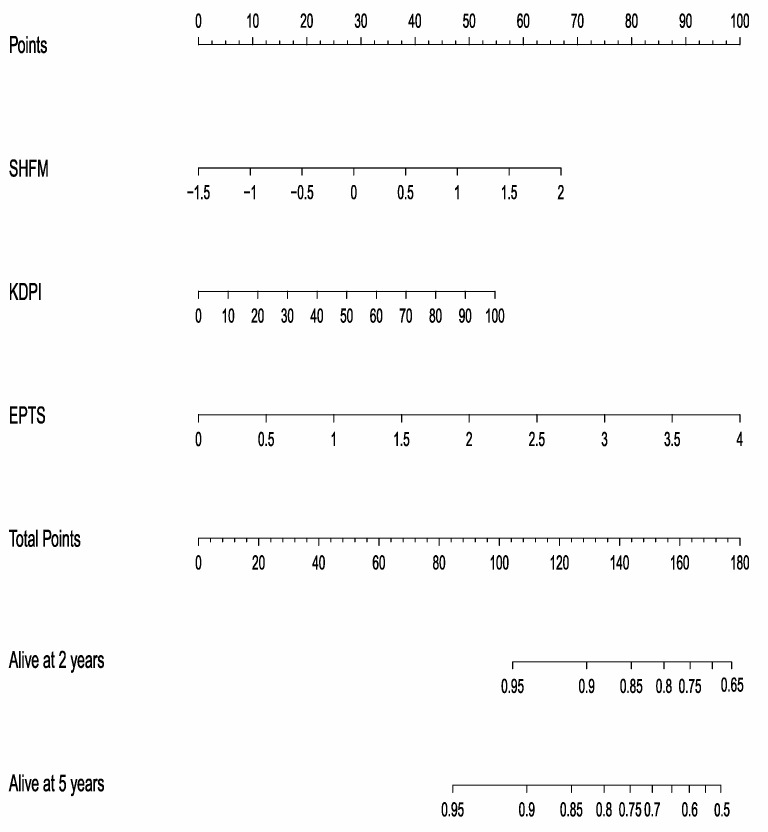
Prediction nomogram for 2-year and 5-year patient survival, using the adapted Seattle Heart Failure Model (SHFM), Kidney Donor Profile Index (KDPI), and Expected Post-Transplant Survival (EPTS) scores.

We used the three-variable model to construct a prediction nomogram for two-year and five-year survival after transplantation (Figure 1). To use this model, the values for adapted SHFM, KDPI, and EPTS are projected onto the Points axis in order to assign points for each variable, which are summed. The value of Total Points is projected onto the axes for two-year and five-year survival. 

For example, a patient whose SFHM = 1.5 and who has a raw EPTS score of 2.0, receiving a kidney from a donor with KDPI = 80, would have 57 points for the SHFM, 50 points for EPTS, and 45 points for the KDPI, for a total of 152 points. Projecting 152 points onto the survival axes would yield a predicted two-year survival of ~0.81 and predicted five-year survival of ~0.70. 

### 3.4. Low Ejection Fraction and Ventricular Hypertrophy Were Not Associated with Mortality

The SHFM was validated in patients with heart failure and ejection fraction less than 40%. In our cohort, only 10 patients had a left ventricular ejection fraction (LV-EF) less than or equal to 40%, of whom nine survived and one died. Sixteen patients had mildly reduced LV-EF (41–49%), of whom thirteen survived and three died. In our cohort, having reduced or mildly reduced LV-EF was not associated with mortality (HR = 0.61 95% CI = 0.08,4.48, *p* = 0.63 and HR = 1.54, 95% CI = 0.47,5.00, *p* = 0.47). Only 7% of the patients had a history of heart failure. 

We investigated if left ventricular hypertrophy (LVH) correlated with mortality because LVH is the most frequently observed cardiac change in individuals with chronic kidney disease [19], and LVH can lead to diastolic dysfunction and heart failure with preserved ejection fraction [20].

LVH was derived from echocardiography routinely performed during the evaluation for transplant. Our cohort had an overall incidence of 69.4% for LVH, which did not differ between survivors (69.2%) and those who died (71.4%). LVH was classified as borderline/mild (44.6%), moderate (21.2%), or severe (4.2%). LVH was not associated with mortality (HR = 1.14, 95% CI = 0.59, 2.21, *p* = 0.7), although the SHFM score was minimally correlated with the presence of LVH (r = 0.11, *p* = 0.04).

### 3.5. SHFM Scores Are Associated with Lower Mortality in Kidney Transplant Patients Than among Heart Failure Patients

The univariate SHFM was used to generate two-year and five-year predictions for the study population as a function of the SHFM score. These were compared to standard two-year and five-year survivals for heart failure patients, as back-calculated from SHFM scores, using the calculations provided in the appendix of the original SHFM paper [9]:

$$\mathrm{Two}-\mathrm{year}\;\mathrm{survival}=e^{\left(-0.0810\times e^{SHFM}\;\right)}$$

$$\mathrm{Five}-\mathrm{year}\;\mathrm{survival}=e^{\left(-0.0810\times e^{SHFM}\;\right)}$$

**Figure 2:**
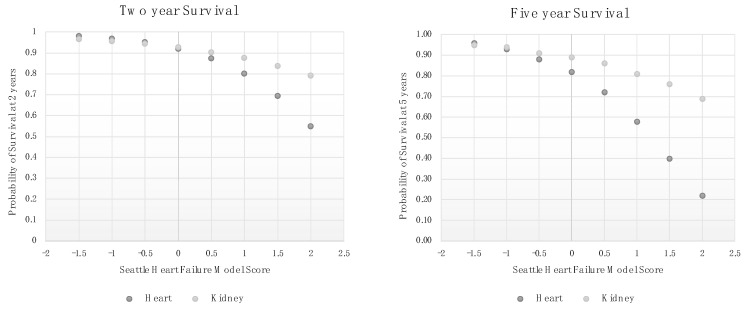
Predicted two and five-year survival according to the Seattle Heart Failure Model (SHFM) for heart failure patients and kidney transplant candidates.

Increasing SHFM was associated with decreased survival in both groups, but the kidney transplant patients had better survivals at all levels of SHFM scores, with increasing divergence as the SFHM increased (Figure 2).

## 4. Discussion

Cardiovascular disease is the leading cause of mortality after kidney transplantation. Despite comprehensive cardiac evaluations, post-transplant cardiac complications continue to be common. There is a need for better cardiac risk models for kidney transplant candidates.

The Seattle Heart Failure Model (SHFM) is a well-established and validated multivariable risk model for predicting survival in heart failure patients. The SHFM does not incorporate kidney function as one of its components, so it is one of the few available indices that could be potentially used to assess cardiac risk in patients with kidney disease during the transplant evaluation process. Most of the component variables are already collected during the transplant evaluation, but the model has the additional advantage of assigning weights to each covariate, based on prior trial results. The knowledge gap arises from the fact that the coefficients for the SHFM were established for a different patient population. Therefore, it was necessary to investigate our hypothesis that the SHFM results would be associated with patient survival in the kidney transplant setting.

Many variables included in the SHFM have proven to have significant impact on mortality among patients with ESRD and kidney transplant recipients, including age, coronary artery disease, low ejection fraction, hypertension, hyponatremia, anemia, and hyperuricemia [11,12,13,14,15,16,17]. In our cohort, we found that age, sodium, cholesterol, and lymphocyte count had significant univariate associations with mortality. 

Hyponatremia (serum sodium < 135 mg/dL) is a risk factor for mortality in chronic hemodialysis patients, independently of fluid overload and malnutrition [21]. In our cohort, patients who died had more hyponatremia than patients who survived; however, the hyponatremia was generally mild and clinically insignificant.

Notably, total cholesterol had an inverse association with mortality, which is counterintuitive to results in the general population but consistent with the existing literature regarding the confounding effects of inflammation and/or malnutrition in advanced kidney disease [22]. 

While the SHFM was primarily designed for a heart failure population with significantly reduced ejection fraction, it has also been tested in patients with heart failure and preserved EF, although with a slightly lower predictive ability [10]. The shared risk factors between heart failure and kidney failure incorporated into the SHFM suggested potential applicability to kidney transplant candidates who do not typically have heart failure. 

The Estimated Post-Transplant Survival (EPTS) model is designed to predict patient survival after receiving a kidney transplant, considering age, dialysis vintage, previous solid organ transplantation, and the presence or absence of diabetes. Since 2014, the allocation system has been structured to match KDPI to EPTS in order to allocate high-longevity kidneys with recipients who are expected to have longer post-transplant survival [23]. Both indices would be expected to correlate with patient survival in this cohort because most of the transplants were performed after 2014. We found that the SHFM, EPTS, and KDPI were all independently associated with transplant recipient survival. Our findings highlight the importance of considering cardiac risk when accepting high-KDPI kidneys for high-EPTS patients.

It is important to note that severely reduced ejection fraction (LV-EF < 30%) is a contraindication for kidney transplant [24]. Patients who undergo kidney transplantation are subject to a comprehensive cardiac evaluation and are carefully selected for good cardiac function. Our cohort was therefore typical of a population that is far more homogeneous (and far healthier) than the general kidney failure patient population. Therefore, there was no difference in the LV-EF between those who survived and those who did not. Normally, LV-EF is an excellent predictor of mortality, but in this case, its similarity across both groups did not grant any association with mortality.

Left ventricular hypertrophy (LVH) is present in 60–75% of individuals with chronic kidney disease prior to the need for dialysis and increases to 90% after the initiation of dialysis [19]. The severity and persistence of LVH are strongly associated with mortality risk and cardiovascular events in ESRD patients [25]. Although the incidence of LVH in our cohort was similar to that reported in the literature, our study found that LVH alone was not associated with mortality. This may reflect a reversal of ventricular remodeling that has been observed after successful kidney transplantation [26,27]. Another possible explanation for the lack of association between LVH and mortality could be that our analysis was based on a single measurement before transplant. Serial echocardiographic monitoring might offer more robust prognostic insights [28].

Another advantage of the SHFM is that it incorporates treatment interventions that potentially impact cardiac mortality, such as angiotensin-converting enzyme inhibitors, angiotensin receptor blockers, beta-blockers, and aldosterone blockers. However, patients with ESRD may not always be able to receive guideline-directed therapy for heart failure, due to hypotension, hyperkalemia, and other complications of the uremic state [29]. Interventions targeting SHFM risk factors (e.g., medications for hypertension, heart failure) may or may not improve post-transplant survival; further work will be required to determine whether aggressive pre-transplant risk factor intervention affects the utility of the SHFM score. For example, the use of statins has been shown to significantly reduce cardiovascular mortality after kidney transplant [30], but benefits have not been demonstrated among ESRD patients [31]. The statin variable was ultimately removed from the model by the SHFM investigators, as well, due to the negative results in heart failure patients.

The use of the SHFM in transplant candidates could also aid in evaluating program performance. The SHFM provides additional information that may help improve the interpretation of expected transplant recipient survival, which could refine the context for post-transplant survival performance.

The strength of this study is the use of a well-characterized, sizeable patient cohort. Certain limitations nonetheless merit discussion. Our study is retrospective and observational, providing associations rather than causation. By necessity, variables were not obtained at a single visit, which could be potentially different from the conditions under which the SHFM is normally employed. The selection of patients from a single transplant center could potentially reduce the generalizability of our findings. 

Another limitation of this study is that our population consisted of patients who survived waitlisting and received kidney transplants, a population that could systematically differ from other evaluated patients who died or were removed from the waitlist. This creates a selection bias, which limits the generalizability of our findings. Consequently, further evaluation of the SHFM’s performance across the broader ESRD population would be useful.

Our study design did not account for the impact of post-transplant clinical events on patient survival, and additional unidentified confounders could have potentially influenced our results.

Nonetheless, our results provide preliminary evidence that the SHFM score has strong associations with survival in a kidney transplant population. Prospective studies on larger cohorts, including patients at the time of listing for transplant, could be undertaken to validate the hypothesis that the use of the SHFM could assist clinicians who are evaluating kidney transplant candidates. A simple and inexpensive tool that aids in making more effective decisions, recognizing cardiovascular risk factors, and guiding optimal pre-transplant preparation would be welcome. 

## Figures and Tables

**Table 1:** Clinical characteristics, by survival outcome.

		All	Survived	Died	*p*-Value
	N	N = 360	n = 317	n = 43	
Recipient	Age in years at transplant	52.3 (12.7)	51.4 (12.7)	59.1 (11.4)	<0.001
	Male Gender, %	64.4	65.9	53.5	0.1
	Ethnicity, %				
	African American	68.6	68.5	69.8	0.06
	White	23.6	24.6	16.3	
	Hispanic	5.8	5.7	7.0	
	Asian	2.0	1.3	7.0	
	Body mass index at transplant	28.2 (5.3)	28.1 (5.3)	28.3 (5.4)	0.9
	EPTS, raw score	2.0 (0.7)	2.0 (0.7)	2.4 (0.6)	<0.001
	Ischemic disease, %	18.1	17.4	23.3	0.3
	Left ventricular hypertrophy, %	69.4	69.2	71.4	0.6
	Mildly reduced EF, %	4.4	4.1	6.9	0.6
	Reduced EF, %	2.7	2.8	2.3	0.6
	Smoking history, %	42.9	43.0	41.9	0.9
	Months of dialysis, median (IQR) *	72 (47, 90)	71 (46, 90)	77 (52, 89)	0.4
	Hypertension, %	83.9	83.6	86.1	0.7
	Diabetes, %	38.1	36.0	53.5	0.02
Donor	Kidney Donor Profile Index	54 (23)	53 (23)	62 (25)	0.009
	Hypothermic perfusion, %	42.0	40.3	54.8	0.07
	Donation after cardiac death, %	40.8	41.3	37.2	0.6

Abbreviations: IQR (interquartile range), EF (Ejection Fraction), EPTS (Expected Post-Transplant Survival). Data presented as mean (standard deviation) unless otherwise specified. * Patients transplanted pre-emptively were excluded.

**Table 2:** Univariate hazard ratios for death after kidney transplantation on the SHFM and its individual components.

	HR (95% CI)	*p*-Value
SHFM	1.76 (1.10, 2.83)	0.02
Age, per year	1.05 (1.02, 1.08)	<0.001
Male sex	0.62 (0.34, 1.12)	0.1
NYHA (ref: NYHA class 1)		
Class 2	1.13 (0.35, 3.57)	0.8
Class 3	2.04 (0.28, 14.9)	0.4
Ischemic	1.38 (0.68, 2.79)	0.4
LV ejection fraction, per 10%	1.02 (0.71, 1.47)	0.9
Systolic BP, per 10 mm Hg	1.03 (0.90, 1.16)	0.7
Sodium, per 1 mEq/L	0.90 (0.84, 0.96)	0.003
Hemoglobin	0.94 (0.79, 1.11)	0.5
% Lymphocytes	0.96 (0.93, 1.00)	0.04
Uric acid	1.01 (0.87, 1.17)	0.9
Cholesterol, per 20 mg/dL	0.83 (0.70, 0.99)	0.03
Diuretics	1.00 (0.99, 1.01)	0.7
Allopurinol	1.23 (0.38, 4.00)	0.7
Beta blocker	1.05 (0.56, 1.97)	0.9
ARB	0.83 (0.33, 2.10)	0.7
ACE	1.01 (0.50, 2.05)	0.9

Abbreviations: SHFM (Seattle Heart Failure Model), NYHA (New York Heart Association), LV (left ventricular), BP (blood pressure), ARB (angiotensin receptor blocker), ACE (angiotensin-converting enzyme inhibitor).

**Table 3:** Seattle Heart Failure Model and its components, by survival outcome.

	All	Survived	Died	*p*-Value
N	N = 360	n = 317	n = 43	
SHFM score	−0.01 (0.60)	−0.04 (0.59)	0.16 (0.66)	0.04
Age in years at evaluation	51.4 (12.7)	50.5 (12.6)	57.9 (11.6)	<0.001
Male Gender, %	64.4	65.9	53.5	0.1
NYHA class, %				0.9
Class 1	91.1	91.2	90.7	
Class 2	7.0	6.9	7.0	
Class 3	1.9	1.9	2.3	
Ischemic disease, %	18.1	17.4	23.3	0.3
LV ejection fraction	60 (8)	60(8)	60 (8)	0.9
Systolic blood pressure, mm Hg	134 (24)	134 (23)	137 (26)	0.5
Serum sodium, mEq/L	139 (3)	140 (3)	138 (3)	0.008
Hemoglobin, g/dL	11.4 (1.7)	11.4 (1.7)	11.2 (1.9)	0.6
% Lymphocytes	24 (9)	25 (10)	22 (7)	0.01
Serum uric acid, mg/dL	5.7 (1.9)	5.7 (1.9)	5.8 (1.8)	0.8
Serum cholesterol, mg/dL	163 (42)	165 (43)	149 (30)	0.004
Diuretic use, %	20.6	20.5	20.9	0.9
Allopurinol use, %	6.1	6.0	7.0	0.8
Beta blocker use, %	64.4	64.4	65.1	0.9
ARB/ACE use, %	36.9	37.2	34.9	0.7
Statin use, %	41.4	40.7	46.5	0.5

Abbreviations: SHFM (Seattle Heart Failure Model), NYHA (New York Heart Association), LV (left ventricular), ARB (angiotensin receptor blocker), ACE (angiotensin-converting enzyme inhibitor). Data presented as mean (standard deviation) unless otherwise specified.

## Data Availability

The data presented in this study are available on request from the corresponding author.

## Supplementary Materials

The following supporting information can be downloaded at https://www.mdpi.com/article/10.3390/jcm12247614/s1, Supplementary Figure S1: Distribution of the SHFM scores in the kidney transplant cohort; Supplementary data: Calculation of an adapted Seattle Heart Failure Model for kidney transplant candidates with advanced chronic kidney disease or kidney failure.

## References

[1] United States Renal Data (2019). 2018 USRDS annual report: Epidemiology of kidney disease in the United States. Am. J. Kidney Dis..

[2] Awan A.A., Niu J., Pan J.S., Erickson K.F., Mandayam S., Winkelmayer W.C., Navaneethan S.D., Ramanathan V. (2018). Trends in the Causes of Death among Kidney Transplant Recipients in the United States (1996–2014). Am. J. Nephrol..

[3] Gansevoort R.T., Correa-Rotter R., Hemmelgarn B.R., Jafar T.H., Heerspink H.J.L., Mann J.F., Matsushita K., Wen C.P. (2013). Chronic kidney disease and cardiovascular risk: Epidemiology, mechanisms, and prevention. Lancet.

[4] Kottgen A., Russell S.D., Loehr L.R., Crainiceanu C.M., Rosamond W.D., Chang P.P., Chambless L.E., Coresh J. (2007). Reduced Kidney Function as a Risk Factor for Incident Heart Failure: The Atherosclerosis Risk in Communities (ARIC) Study. J. Am. Soc. Nephrol..

[5] House A.A., Wanner C., Sarnak M.J., Piña I.L., McIntyre C.W., Komenda P., Kasiske B.L., Deswal A., de Filippi C.R., Cleland J.G.F. (2019). Heart failure in chronic kidney disease: Conclusions from a Kidney Disease: Improving Global Outcomes (KDIGO) Controversies Conference. Kidney Int..

[6] Lentine K.L., Schnitzler M.A., Abbott K.C., Li L., Burroughs T.E., Irish W., Brennan D.C. (2005). De Novo Congestive Heart Failure After Kidney Transplantation: A Common Condition With Poor Prognostic Implications. Am. J. Kidney Dis..

[7] Corrà U., Agostoni P., Giordano A., Cattadori G., Battaia E., La Gioia R., Scardovi A.B., Emdin M., Metra M., Sinagra G. (2016). The metabolic exercise test data combined with Cardiac And Kidney Indexes (MECKI) score and prognosis in heart failure. A validation study. Int. J. Cardiol..

[8] Sartipy U., Dahlström U., Edner M., Lund L.H. (2014). Predicting survival in heart failure: Validation of the MAGGIC heart failure risk score in 51043 patients from the Swedish Heart Failure Registry. Eur. J. Heart Fail..

[9] Levy W.C., Mozaffarian D., Linker D.T., Sutradhar S.C., Anker S.D., Cropp A.B., Anand I., Maggioni A., Burton P., Sullivan M.D. (2006). The Seattle Heart Failure Model. Circulation.

[10] May H.T., Horne B.D., Levy W.C., Kfoury A.G., Rasmusson K.D., Linker D.T., Mozaffarian D., Anderson J.L., Renlund D.G. (2007). Validation of the Seattle Heart Failure Model in a community-based heart failure population and enhancement by adding B-type natriuretic peptide. Am. J. Cardiol..

[11] den Dekker W.K., Slot M.C., Kho M.M.L., Galema T.W., van de Wetering J., Boersma E., Roodnat J.I. (2020). Predictors of postoperative cardiovascular complications up to 3 months after kidney transplantation. Neth. Hear. J..

[12] Aalten J., Hoogeveen E.K., Roodnat J.I., Weimar W., Borm G.F., de Fijter J.W., Hoitsma A.J. (2008). Associations between pre-kidney-transplant risk factors and post-transplant cardiovascular events and death. Transpl. Int..

[13] Gill J.S., Abichandani R., Kausz A.T., Pereira B.J.G. (2002). Mortality after kidney transplant failure: The impact of non-immunologic factors. Kidney Int..

[14] Ozkul F., Arik M.K., Erbiş H., Akbaş A., Yilmaz V.T., Barutcu A., Osmanoğlu I.A., Kocak H. (2016). Left ventricle ejection fraction may predict mortality in renal transplant patients. Ren. Fail..

[15] Opelz G., Dohler B. (2005). Improved Long-Term Outcomes After Renal Transplantation Associated with Blood Pressure Control. Am. J. Transplant..

[16] Dahle D.O., Jenssen T., Holdaas H., Leivestad T., Vårdal M., Mjøen G., Reisaeter A.V., Toft I., Hartmann A. (2014). Uric acid has a J-shaped association with cardiovascular and all-cause mortality in kidney transplant recipients. Clin. Transplant..

[17] Devine P.A., Courtney A.E., Maxwell A.P. (2019). Cardiovascular risk in renal transplant recipients. J. Nephrol..

[18] Estimated Post Transplant Survival Calculator (2020). Organ Procure Transplant Network. https://optn.transplant.hrsa.gov/resources/allocation-calculators/epts-calculator.

[19] Foley R.N., Curtis B.M., Randell E.W., Parfrey P.S. (2010). Left Ventricular Hypertrophy in New Hemodialysis Patients without Symptomatic Cardiac Disease. Clin. J. Am. Soc. Nephrol..

[20] Gradman A.H., Alfayoumi F. (2006). From Left Ventricular Hypertrophy to Congestive Heart Failure: Management of Hypertensive Heart Disease. Prog. Cardiovasc. Dis..

[21] Dekker M.J., Marcelli D., Canaud B., Konings C.J., Leunissen K.M., Levin N.W., Raimann J.G., van der Sande F.M., Usvyat L.A., Kotanko P. (2016). Unraveling the relationship between mortality, hyponatremia, inflammation and malnutrition in hemodialysis patients: Results from the international MONDO initiative. Eur. J. Clin. Nutr..

[22] Liu Y., Coresh J., Eustace J.A., Longenecker J.C., Jaar B., Fink N.E., Tracy R.P., Powe N.R., Klag M.J. (2004). Association between cholesterol level and mortality in dialysis patients: Role of inflammation and malnutrition. JAMA.

[23] Friedewald J.J., Samana C.J., Kasiske B.L., Israni A.K., Stewart D., Cherikh W., Formica R.N. (2013). The kidney allocation system. Surg. Clin. N. Am..

[24] Chadban S.J.B., Ahn C., Axelrod D.A.M., Foster B.J.M., Kasiske B.L., Kher V.M., Kumar D.M., Oberbauer R., Pascual J., Pilmore H.L. (2020). KDIGO Clinical Practice Guideline on the Evaluation and Management of Candidates for Kidney Transplantation. Transplantation.

[25] Zoccali C., Benedetto F.A., Mallamaci F., Tripepi G., Giacone G., Stancanelli B., Cataliotti A., Malatino L.S. (2004). Left ventricular mass monitoring in the follow-up of dialysis patients: Prognostic value of left ventricular hypertrophy progression. Kidney Int..

[26] Hewing B., Dehn A.M., Staeck O., Knebel F., Spethmann S., Stangl K., Baumann G., Dreger H., Budde K., Halleck F. (2016). Improved Left Ventricular Structure and Function after Successful Kidney Transplantation. Kidney Blood Press. Res..

[27] Zapolski T., Furmaga J., Wysokiński A.P., Wysocka A., Rudzki S., Jaroszyński A. (2019). The atrial uremic cardiomyopathy regression in patients after kidney transplantation—The prospective echocardiographic study. BMC Nephrol..

[28] Foley R.N., Parfrey P.S., Kent G.M., Harnett J.D., Murray D.C., Barre P.E. (2000). Serial change in echocardiographic parameters and cardiac failure in end-stage renal disease. J. Am. Soc. Nephrol..

[29] Heywood T., Fonarow G. (2007). High prevalence of renal dysfunction and its impact on outcome in 118,465 patients hospitalized with acute decompensated heart failure: A report from the adhere database. J. Card. Fail..

[30] Navaneethan S.D., Perkovic V., Johnson D.W., Nigwekar S.U., Craig J.C., Strippoli G.F. (2014). HMG CoA reductase inhibitors (statins) for kidney transplant recipients. Cochrane Database Syst. Rev..

[31] Palmer S.C., Navaneethan S.D., Craig J.C., Johnson D.W., Perkovic V., Nigwekar S.U., Hegbrant J., Strippoli G.F. (2013). HMG CoA reductase inhibitors (statins) for dialysis patients. Cochrane Database Syst. Rev..

