# Supplementary material for: The Seattle Heart Failure Model in Kidney Transplant Recipients
Source: J Clin Med. 2023 Dec 11;12(24):7614. doi: 10.3390/jcm12247614 (PMC10743453; doi:10.3390/jcm12247614)
Supplement: Supplementary file 1 [file jcm-12-07614-s001.zip › jcm-2691259-supplementary.pdf]

Supplementary data

Calculation of an adapted Seattle Heart Failure Model for kidney transplant candidates with advanced chronic kidney disease or kidney failure

(Adapted from Levy et al. Circulation 2006, pp. 1424-1433)

The score resulted from summation of points assessed for the following covariates:

Age: Age in years on the date of evaluation was divided by 10, and multiplied by ln(1.090)

Sex: Male sex was assigned ln(1.089)=0.08526 points.

Female sex was assigned 0 points.

New York Heart Association: Categorical class (1-4) was multiplied by ln(1.600)

Ejection fraction: (100/ejection fraction) was multiplied by ln(l.030)

Clinical ischemia: ln(1.354) points were assigned for clinical ischemia, defined as: history of coronary artery disease or myocardial infarction

Systolic BP: Systolic blood pressure was divided by ten, and multiplied by ln(0.877)

Diuretic use: Diuretic exposure was standardized as "furosemide equivalents"

Conversion of bumetanide: mg x 27

Conversion of HCTZ/chlorthalidone: mg x 3.2

Conversion of torsemide mg x 2

Doses were expressed per kg body weight

Allopurinol use: Assigned ln(1.571) points if allopurinol in use.

Serum sodium (Na):Zero points assigned if [Na+]≥ 138 mEq/L

Otherwise (138-Na) multiplied by ln(1.050)

Serum cholesterol: (100/cholesterol) multiplied by ln(2.206)

Hemoglobin (Hgb): Zero points if Hgb=16

If Hgb>16: (Hgb-16) multiplied by ln(1.336)

If Hgb<16: (16-Hgb) multiplied by ln(1.124)

<u>% lymphocytes in peripheral blood leukocytes (%Lymph)</u>:

If <47% lymphocytes, (%Lymph/5) multiplied by ln(0.897)

If ≥47% lymphocytes, (47/5) multiplied by ln(0.8997)

<u>Serum uric acid (UA)</u>:

If UA>3.4, UA multiplied by ln(1.064)

If UA≤3.4, 3.4 multiplied by ln(1.064)

<u>Use of angiotensin converting enzyme inhibition (ACE)</u>:

Ln(0.77) points assigned for use

<u>Use of angiotensin recepter blocking agents (ARB)</u>:

Ln(0.85) points assigned for use

<u>Use of beta blockers (BB)</u>:

Ln(0.66) points assigned for use

<u>Use of K-sparing diuretics (Ksp)</u>:

Ln(0.74) points assigned for use

1. Note is made that Hgb>16, and use of K-sparing diuretics are both very uncommon in a kidney transplant candidate population.
2. Updates were made to the original SHFM model, with removal of the variable for statins. "Given the negative results with the addition of statins in large randomized clinical trials". We removed it from our adapted SHFM equation as well.
3. The updated equations include variables for vasopressors and advanced heart failure device therapy. No kidney transplant candidates had any of these infusions.

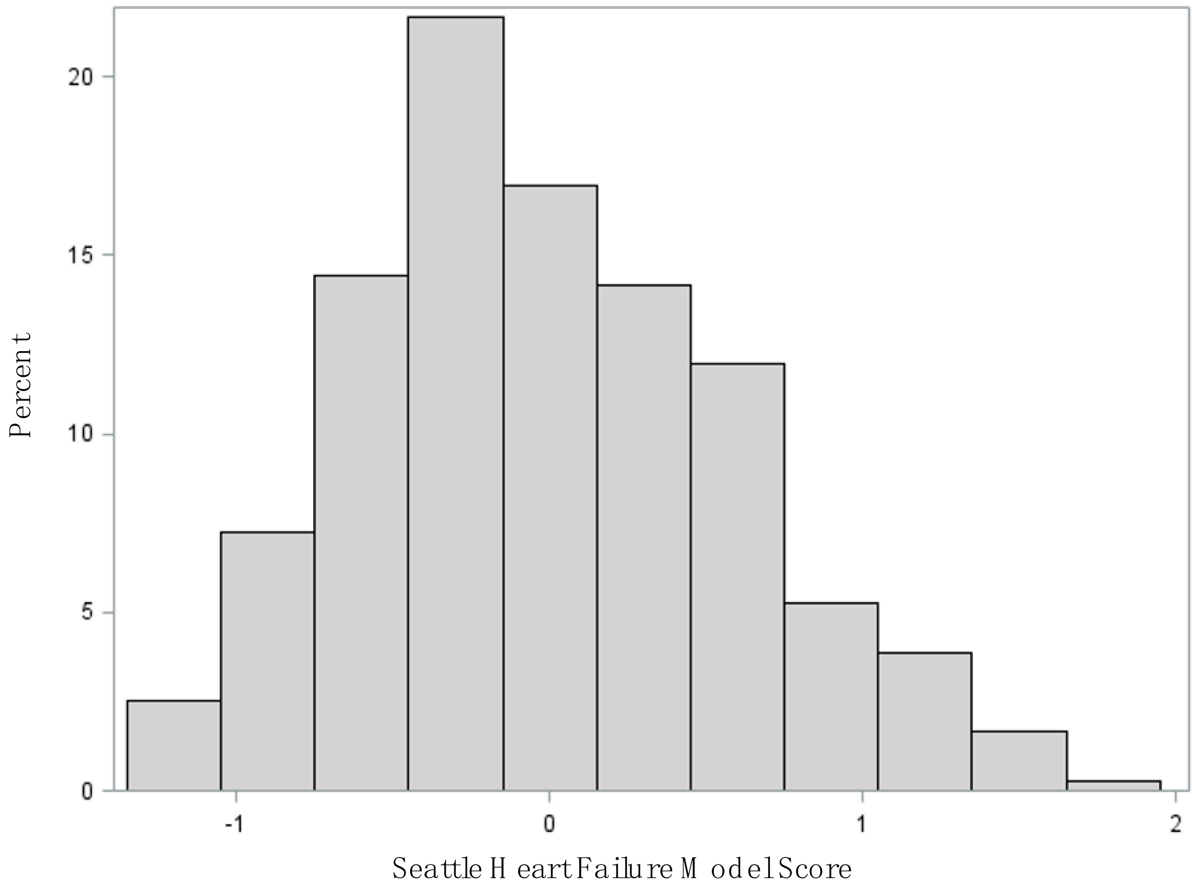

Figure S1: Distribution of the SHFM scores in the kidney transplant cohort
